# Extracorporeal Life Support in ARDS: Lessons from Maternal–Fetal Physiology

**DOI:** 10.3390/arm94050065

**Published:** 2026-09-07

**Authors:** Raffaele Merola, Denise Battaglini, Sung-Min Cho

**Affiliations:** 1 Anesthesia and Intensive Care Medicine, Department of Critical Care, AORN Ospedali Dei Colli, 80131 Naples, Italy; 2 Anesthesia and Intensive Care, IRCCS AOM, Ospedale Policlinico San Martino, 16132 Genoa, Italy; 3 Department of Surgical Sciences and Integrated Diagnostics, University of Genoa, 16132 Genoa, Italy; 4 Division of Neuroscience Critical Care, Departments of Neurology, Surgery, and Anesthesiology and Critical Care Medicine, Johns Hopkins University School of Medicine, Baltimore, MD 21287, USA

**Keywords:** acute respiratory distress syndrome, extracorporeal life support, extracorporeal membrane oxygenation, extracorporeal carbon dioxide removal, physiology

## Abstract

**Highlights:**

**What are the main findings?**

- The maternal–fetal circulation exemplifies how sustained extracorporeal gas exchange can be integrated into systemic physiology while minimizing the mechanical and metabolic burden on the native lung.
- This biological paradigm supports reconceptualizing ECLS in severe ARDS as a transition toward an extracorporeal-centered physiological state rather than simply an escalation of conventional respiratory support.

**What are the implications of the main findings?**

- A physiology-oriented approach to ECLS may shift therapeutic priorities from normalization of arterial blood gases toward coordinated lung and cardiopulmonary unloading through the integration of extracorporeal flow, ventilation, and cardiovascular management.
- Future trials should incorporate physiological criteria for patient selection, treatment titration, and response assessment, alongside outcomes reflecting cardiopulmonary protection and functional recovery.

**Abstract:**

Despite decades of clinical experience, the physiological rationale for extracorporeal life support (ECLS) in acute respiratory distress syndrome (ARDS) remains incompletely defined. Existing trials have primarily evaluated ECLS as a rescue intervention, with limited attention to the physiological adaptations required to sustain extracorporeal gas exchange. We propose that the maternal–fetal circulation, the only naturally occurring example of prolonged extracorporeal gas exchange, provides a hypothesis-generating physiological analogy. In this paradigm, gas exchange is externalized through a low-resistance, high-flow circuit while systemic physiology reorganizes to unload the native lung. Translating this concept to severe ARDS suggests that ECLS should be viewed not simply as an adjunct to conventional support, but as a transition to an alternative physiological state centered on extracorporeal gas exchange. This perspective may inform physiology-based patient selection, management strategies, and the design of future clinical trials.

## 1. Introduction

After more than five decades of clinical experience and several randomized controlled trials, the role of extracorporeal life support (ECLS) in severe acute respiratory distress syndrome (ARDS) remains incompletely defined [1,2]. Large studies have demonstrated the feasibility and safety of ECLS, yet they have not consistently shown a reproducible survival benefit across broadly defined ARDS populations [3,4,5,6]. Beyond clinical outcomes, however, a more fundamental issue remains unresolved: the physiological rationale for extracorporeal support in ARDS has largely been assumed rather than explicitly articulated or rigorously tested.

This gap may reflect not only limitations of the intervention itself, but also the conceptual framework within which it has been evaluated. ARDS encompasses a heterogenous spectrum of biological, morphological, and physiological phenotypes, yet ECLS has largely been studied as a uniform, binary exposure [1,7,8]. Randomized trials have primarily focused on whether extracorporeal support improves survival, while giving comparatively little attention to which physiological functions are being substituted, under what conditions this substitution is most relevant and how the organism adapts to extracorporeal support as a systemic level [3,4,9].

Notably, although previous randomized trials have incorporated specific physiological considerations, none has directly tested the broader hypothesis that extracorporeal support should be implemented as a coordinated physiological reconfiguration aimed at unloading the native lung.

Although the European Society of Intensive Care Medicine (ESICM) ARDS and Extracorporeal Life Support Organization (ELSO) guidelines provide a rigorous and transparent synthesis of the available evidence, their recommendations regarding veno-venous extracorporeal membrane oxygenation (VV-ECMO) and extracorporeal carbon dioxide removal (ECCO_2_R), which differ substantially in extracorporeal blood flow, gas-exchange capacity, and physiological effects, remain inherently limited by the way ECLS has historically been conceptualized [10,11]. In most studies, ECLS has been evaluated as a relatively uniform, late-stage rescue therapy and primarily assessed through short-term mortality endpoints [10]. Such approach insufficiently captures the profound biological and physiological heterogeneity inherent to ARDS, the temporal dynamics of ventilator-induced lung injury (VILI), variability in background lung-protective strategies, differences in center-specific expertise, and the ethical and methodological challenges introduced by crossover and rescue study designs, thereby oversimplifying an intervention whose effects are highly context-dependent and fundamentally rooted in physiology.

Re-examining the rationale for extracorporeal support in ARDS therefore requires a return to first principles. In this regard, the maternal–fetal circulation represents the most complete and evolutionarily validated example of sustained extracorporeal gas exchange in mammals.

## 2. Maternal–Fetal Circulation as a Model of Extracorporeal Gas Exchange

In fetal life, pulmonary gas exchange is functionally absent. Oxygen uptake and carbon dioxide elimination occur entirely through the placenta; an extracorporeal interface interposed between two distinct circulations. Importantly, placental gas exchange operates under conditions that would be considered profoundly hypoxemic by postnatal standards: fetal arterial oxygen tension typically remains below 40 mmHg. Despite these low oxygen tensions, adequate oxygen delivery is maintained through a combination of physiological adaptations, including high uteroplacental blood flow, elevated hemoglobin concentration, the increased oxygen affinity of fetal hemoglobin, and the large diffusive surface area [12]. This arrangement underscores a fundamental physiological distinction between oxygen tension and oxygen delivery. Placental gas exchange is not designed to normalize arterial partial pressures, but rather to sustain convective transport of oxygen at low pressure gradients.

From a hemodynamic standpoint, placental gas exchange operates without high pressure gradients. Uteroplacental blood flow reaches approximately 500–700 mL/min at term—representing roughly 10–15% of maternal cardiac output—and is sustained predominantly through profound reductions in vascular resistance rather than increases in driving pressure. This high-flow, low-pressure circulation results from structural remodeling of the uterine vasculature. Consequently, flow is governed predominantly by changes in vascular geometry rather than kinetic energy transfer, consistent with the principles described by Poiseuille’s law [13,14]. Crucially, the placental circulation functions as a low-resistance shunt that assumes the burden of gas exchange without imposing significant afterload on the maternal heart or mechanical stress on the maternal lungs [15].

## 3. Systemic Adaptations Supporting Extracorporeal Physiology

The effectiveness of fetal extracorporeal gas exchange is inseparable from extensive maternal physiological adaptation. Pregnancy is characterized by a 30–50% increase in circulating blood volume, a substantial rise in cardiac output, and a marked reduction in systemic and pulmonary vascular resistance. These changes preserve efficient right ventricular–pulmonary arterial coupling despite the increase in preload associated with expanded circulatory volume [14].

Respiratory physiology is similarly reorganized. Minute ventilation increases by approximately 40%, largely through increased tidal volume, resulting in a sustained reduction in PaCO_2_ to around 30 mmHg. This chronic respiratory alkalosis enhances the transplacental CO_2_ gradient and facilitates oxygen transfer while avoiding excessive mechanical stress on the maternal lungs [16].

Taken together, these adaptations illustrate a consistent physiological principle: when gas exchange must be externalized, biological systems do not compensate by increasing pressure, mechanical load, or organ strain. Instead, they establish a parallel, low-resistance, high-flow circuit that assumes the burden of gas exchange while deliberately unloading the non-functional lung.

## 4. Translating the Maternal–Fetal Paradigm to Severe ARDS

When translated into clinical terms, this paradigm offers potentially important insights for severe ARDS. In this analogy, the patient with ARDS resembles the fetus, whose lung is unable to sustain effective gas exchange, while the extracorporeal circuit assumes a role analogous to the maternal circulation. Notably, during pregnancy the fetus does not adapt to maternal physiology; rather, maternal physiology is extensively reorganized to support extracorporeal gas exchange on behalf of the fetus [13,14,15,16].

Most randomized trials evaluating extracorporeal support in ARDS have implicitly assumed that systemic physiology should remain largely unchanged following ECLS initiation, effectively studying extracorporeal gas exchange as an adjunct rather than as a transition to a distinct physiological state [3,4,5,6,9]. In this framework, extracorporeal support is introduced into an otherwise conventional cardiopulmonary system and expected to integrate without requiring major systemic adaptation, an assumption that may be fundamentally discordant with the biological principles governing sustained extracorporeal respiration.

The maternal–fetal model suggests a different hierarchy. Once extracorporeal gas exchange becomes necessary, the supporting circulation may need to be actively configured to optimize flow, resistance, and cardiopulmonary coupling within the extracorporeal circuit, with the explicit objective of unloading the injured lung.

Clinically, this paradigm implies that initiation of extracorporeal support should trigger a deliberate shift in therapeutic priorities. Rather than maintaining conventional ventilatory targets and hemodynamic norms while adding extracorporeal gas exchange, ECLS should prompt an intentional unloading strategy, in which ventilatory settings, extracorporeal flows, and cardiovascular management are reoriented to minimize lung stress, pulmonary vascular load, and right ventricular work, even at the expense of accepting non-physiological arterial blood gas values. Within this framework, the primary therapeutic goals shift from normalization of arterial oxygen and carbon dioxide tensions to indices of cardiopulmonary protection, including transpulmonary and driving pressures, mechanical power, pulmonary vascular resistance, and right ventricular–pulmonary arterial coupling—parameters that remain largely unaddressed in current clinical guidelines [10,11]. The absence of such a physiological reorientation may help explain the variable results of randomized trials, in which extracorporeal gas exchange was frequently superimposed onto conventional management strategies without a parallel restructuring of systemic physiology [3,4,5,6,9].

This analogy, however, should be interpreted with caution. The placenta is a highly specialized biological interface, whereas adult ECLS relies on artificial circuits with inherent technical and physiological constraints. Moreover, unlike the coordinated and predictable adaptations of pregnancy, ARDS is a heterogeneous and dynamically evolving condition, making the translation of this principle to adult ECLS necessarily indirect and hypothesis-generating.

## 5. Implications for Future Trials and Clinical Practice

**Figure 1:**
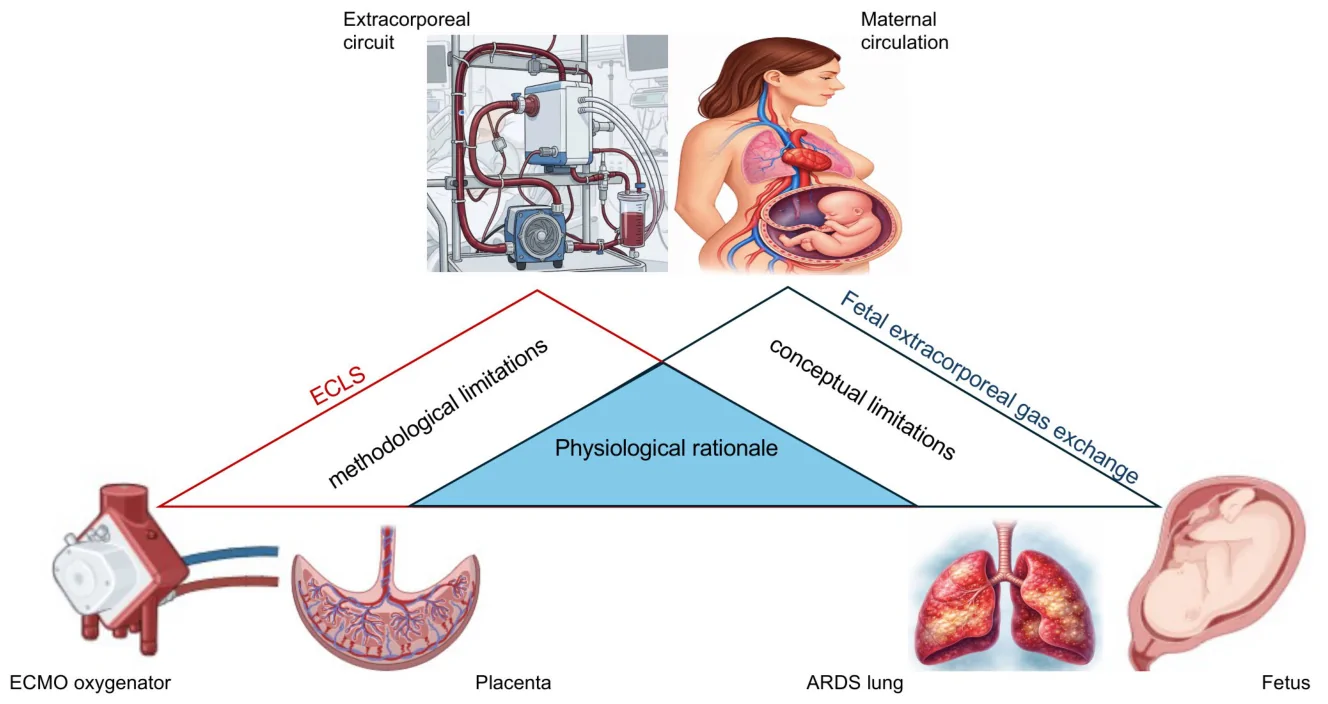
**Conceptual analogy between maternal–fetal physiology and extracorporeal life support in severe ARDS.** In maternal–fetal circulation, the placenta serves as the extracorporeal gas-exchange interface, while the maternal circulation provides systemic flow to sustain this interface, enabling oxygen delivery and carbon dioxide removal for the fetus. In severe ARDS, the ECMO oxygenator parallels the placenta as the extracorporeal gas-exchange organ, and the extracorporeal circuit functions analogously to the maternal circulation by providing a high-flow, low-resistance pathway. The injured ARDS lung, in turn, resembles the fetus, whose native lungs are incapable of effective gas exchange. Although this analogy is grounded in physiologic principles, important conceptual and methodological limitations remain and should be considered when interpreting extracorporeal support in ARDS. **Abbreviations:** ARDS, acute respiratory distress syndrome; ECMO, extracorporeal membrane oxygenation; ECLS, extracorporeal life support.

Viewed through this lens, the limitations of existing trials appear as much conceptual as methodological (Figure 1). Patient selection has often overlooked ARDS subphenotypes with distinct physiological responses [17,18]. Trials have been conducted across centers with heterogeneous expertise, despite the dependence of ECLS outcomes on nuanced management of flow, ventilation, and cardiopulmonary interactions. Future studies could therefore incorporate physiological criteria not only for patient selection, but also for treatment titration and assessment of response [CUrrent Practices of Intensive Care for the Management of Acute Respiratory Distress Syndrome in EurOpe. (CUPIDO) ClinicalTrials.gov ID NCT06714201]. Sponsor Universitätsklinikum Hamburg-Eppendorf.

Information provided by Markus Haar, University of Hamburg-Eppendorf (Responsible Party). Last Update Posted 3 December 2024]. At the bedside, this approach would shift the decision to initiate ECLS from refractory hypoxemia alone toward evidence of physiological burden—such as rising transpulmonary pressure, mechanical power, or right ventricular strain—and would support reassessment of the response after extracorporeal support is established [8,17,19,20,21,22,23,24,25]. Although several randomized trials incorporated physiological eligibility criteria and management targets, these variables have seldom been evaluated as explicit determinants of extracorporeal support initiation or as mechanistic mediators of treatment effect. Correspondingly, trial outcomes largely centered on short-term mortality, with comparatively little attention to indices of organ protection, cardiopulmonary recovery, and long-term functional status [3,4,5,6,9].

Applied clinically, the maternal–fetal paradigm generates a testable hypothesis: in selected patients with severe ARDS, outcomes may improve when extracorporeal support is coupled with proactive cardiovascular and ventilatory reconfiguration aimed at optimizing extracorporeal flow and minimizing pulmonary stress, rather than preserving conventional cardiopulmonary targets. The maternal–fetal system represents a biologically validated example of sustained extracorporeal gas exchange and of the systemic adaptations required to support it, despite its fundamental differences from adult cardiopulmonary physiology.

Reconceptualizing ECLS as a transition to an alternative, extracorporeal-centered physiology—rather than merely a rescue adjunct for failing lungs—may be pivotal to realizing its full therapeutic potential in ARDS.

Future investigations may benefit from an explicitly physiology-based design: focusing on well-characterized subphenotypes, conducted in experienced centers, initiated according to predefined physiological criteria and evaluated using composite outcomes that capture organ protection and functional recovery.

## 6. Conclusions

ECLS may be more appropriately understood as a transition to an alternative physiological state, in which gas exchange is deliberately externalized to protect a lung that is temporarily unable to sustain its function. The maternal–fetal circulation exemplifies a fundamental biological principle: when extracorporeal gas exchange becomes essential, systemic physiology reorganizes around the extracorporeal interface, while the native lung is purposefully unloaded. Translating this principle to ARDS likely requires a corresponding shift in clinical thinking—from compelling the injured lung to meet metabolic demands to deliberately shaping extracorporeal physiology around the needs of the patient. The central hypothesis is that re-centering physiology as the primary framework for patient selection, timing of initiation, and management may allow ECLS to move beyond inconsistent trial results and toward a more individualized and mechanistically grounded strategy for ARDS. Whether this physiology-driven strategy can improve cardiopulmonary protection and clinical outcomes remains to be established in prospective trials.

## Data Availability

No new data were created or analyzed in this study. Data sharing is not applicable to this article.
